# Overexpression of YB1 C‐terminal domain inhibits proliferation, angiogenesis and tumorigenicity in a SK‐BR‐3 breast cancer xenograft mouse model

**DOI:** 10.1002/2211-5463.12004

**Published:** 2016-01-11

**Authors:** Jian‐hong Shi, Nai‐peng Cui, Shuo Wang, Ming‐zhi Zhao, Bing Wang, Ya‐nan Wang, Bao‐ping Chen

**Affiliations:** ^1^ Central LaboratoryHebei Laboratory of Mechanism and Procedure of Cancer Radiotherapy and ChemotherapyAffiliated Hospital of Hebei UniversityBaodingChina; ^2^ Department of OncologyAffiliated Hospital of Hebei UniversityBaodingChina; ^3^ Department of PathologyAffiliated Hospital of Hebei UniversityBaodingChina

**Keywords:** angiogenesis, breast cancer, C‐terminal domain, proliferation, SK‐BR‐3, Y‐box‐binding protein 1

## Abstract

Y‐box‐binding protein 1 (YB1) is a multifunctional transcription factor with vital roles in proliferation, differentiation and apoptosis. In this study, we have examined the role of its C‐terminal domain (YB1 CTD) in proliferation, angiogenesis and tumorigenicity in breast cancer. Breast cancer cell line SK‐BR‐3 was infected with GFP‐tagged YB1 CTD adenovirus expression vector. An 3‐(4,5‐dimethylthiazol‐2‐yl)‐5‐(3‐carboxymethoxyphenyl)‐2‐(4‐sulfophenyl)‐2H‐tetrazolium (MTS) proliferation assay showed that YB1 CTD decreased SK‐BR‐3 cell proliferation, and down‐regulated cyclin B1 and up‐regulated p21 levels in SK‐BR‐3 cells. YB1 CTD overexpression changed the cytoskeletal organization and slightly inhibited the migration of SK‐BR‐3 cells. YB1 CTD also inhibited secreted VEGF expression in SK‐BR‐3 cells, which decreased SK‐BR‐3‐induced EA.hy926 endothelial cell angiogenesis *in vitro*. YB1 CTD overexpression attenuated the ability of SK‐BR‐3 cells to form tumours in nude mice, and decreased *in vivo* VEGF levels and angiogenesis in the xenografts in SK‐BR‐3 tumour‐bearing mice. Taken together, our findings demonstrate the vital role of YB1 CTD overexpression in inhibiting proliferation, angiogenesis and tumorigenicity of breast cancer cell line SK‐BR‐3.

**Abbreviations**
CCL5: Chemokine ligand 5
CSD: cold‐shock domain
CTD: C‐terminal domain
DAPI: 4′,6‐diamidino‐2‐phenylindole
DMEM: Dulbecco's Modified Eagle's Medium
FBS: Fetal bovine serum
MMP‐13: Matrix metalloproteinase‐13
MTS: 3‐(4,5‐dimethylthiazol‐2‐yl)‐5‐(3‐carboxymethoxyphenyl)‐2‐(4‐sulfophenyl)‐2H‐tetrazolium
VEGF: vascular endothelial growth factor
VSMCs: vascular smooth muscle cell
YB1: Y‐box‐binding protein 1

Breast cancer is the most common type of cancer in women with high incidence and mortality, which is mostly due to rapid tumour progression and metastatic spread 1. Like the majority of solid tumours, breast cancer requires the growth of new blood vessels in order to provide itself with continuous supply of oxygen and nutrients. This necessity for new blood vessel formation in turn could be used as an anticancer treatment strategy and in developing new therapies directed against tumour vasculature 2.

Angiogenesis is the formation of new blood vessels from pre‐existing vasculature, which is controlled by proangiogenic factors (such as vascular endothelial growth factor [VEGF], platelet‐derived growth factor [PDGF] and epidermal growth factor [EGF]) and antiangiogenic factors (such as angiostatin and endostatin) 3, 4. Indeed, the VEGF signal‐transduction pathway has been implicated in breast cancer pathogenesis, and anti‐VEGF therapy acting on tumour vasculature has been used for breast cancer therapy 5.

Y‐box‐binding protein 1 (YB1) is a member of the conserved cold‐shock domain (CSD) family; and participates in proliferation, differentiation, metastasis and drug resistance 6. Although the role of YB1 in tumorigenesis has been explored over the years in various models, it is still far from being completely deciphered. It has been shown that YB1 acts as an oncogene with important functions in tumour proliferation and progression 7, 8, 9, 10, 11, 12. YB1 also exhibits pleiotropic functions in promoting different cellular processes like migration and invasion 13, 14, 15. Human YB‐1 protein consists of three functional domains: alanine‐ and proline‐rich N‐terminal A/P domain, highly conserved cold‐shock domain (CSD), and C‐terminal domain (CTD) 16, 17. YB1 binds to 5′‐CTGATTGG‐3′ motif (so‐called Y‐box) or inverted CCAAT box‐binding domain via CSD domain, and regulates the expression of many different genes 18. Furthermore, in our previous studies, we have demonstrated that YB1 interacts with GGGCGG motif (known as GC box) via CTD domain in vascular smooth muscle cell (VSMC) proliferation and differentiation‐related gene promoter, which promotes VSMC differentiation 19, 20. In addition, VSMC and vascular endothelial cells are the most important components of vasculature and tumour angiogenesis 21. Therefore, in this study, we sought to further investigate the role of YB1 CTD in breast cancer cell proliferation, induced endothelial cell tube formation, as well as angiogenesis in mouse model.

## Materials and methods

### Cell culture and cell lines

Human breast cancer cell line SK‐BR‐3 and human umbilical vein cell line EA.hy926 were obtained from the Cell Resource Center of Shanghai Institutes for Biological Sciences, Shanghai, China. SK‐BR‐3 and EA.hy926 cells were maintained in Dulbecco's Modified Eagle's Medium (DMEM) with 10% FBS in a humidified atmosphere with 5% CO_2_ at 37 °C.

### Adenovirus expression vector construction

The GFP‐YB1 CTD fusion protein expression plasmid was generated by inserting the human YB1 CTD (amino acids 125–324) coding region into a pEGFP vector (Clontech Laboratories, Palo Alto, CA, USA) using primers 5′‐tgacaagcttcacaggtcctggtggtgttcc‐3′ (sense) and 5′‐tgacggatccttactcagccccgccctgc‐3′ (antisense). For adenovirus expression vector construction, coding region of GFP or GFP‐YB1 CTD fusion protein (harbouring GFP and human YB1‐coding region for amino acids 125–324) 20 were cloned into the pAD/CMV/V5‐DEST vector (Invitrogen, Carlsbad, CA, USA) to create adenovirus Ad‐GFP or Ad‐GFP‐YB1 CTD using the following primers: 5′‐caccatggtgagcaagggcgaggag‐3′ (sense) and 5′‐ ttactcagccccgccctgctcagcct‐3′ (antisense). Resulting constructs were packaged in 293A cells (Invitrogen) by transfection with Lipofectamine 2000 (Invitrogen) according to manufacturer's instructions.

### Small interfering RNA (siRNA) transfection

Small interfering RNA targeting YB1 (si‐YB1) were purchased from Santa Cruz Biotechnology (Santa Cruz, CA, USA). Transfection was performed using Lipofectamine reagent (Invitrogen) following the manufacturer's instructions. Cells were harvested 48 h after transfection and followed by MTS proliferation analysis and western blotting.

### Western blotting

For western blotting analysis, crude proteins (100 μg) were extracted from SK‐BR‐3 breast cancer cells, resolved by SDS/PAGE and transferred onto a polyvinylidene difluoride membrane (Millipore, Billerica, MA, USA). Membranes were blocked with 5% milk in Tris‐buffered saline with Tween 20 for two hours at 37 °C; and then, incubated overnight at 4 °C with the following primary antibodies: rabbit anti‐YB1 (ab76149, Abcom, Cambridge, MA, USA), rabbit anti‐p21 (sc‐397, Santa Cruz), rabbit anti‐cyclin B1 (1495‐1, Epitomics, CA, USA), rabbit anti‐cyclin D1 (2261‐1, Epitomics), mouse anti‐GFP (sc‐390394, Santa Cruz) or mouse anti‐β‐actin (sc‐47778, Santa Cruz). After incubation with the appropriate secondary antibody [goat anti‐(mouse IgG‐HRP), sc‐2005, Santa Cruz or goat anti‐(rabbit IgG‐HRP), sc‐2004, Santa Cruz], immunoreactive signal of antibody‐antigens was visualized using the Immobilon^™^ Western Chemiluminescent HRP Substrate (Millipore Corporation).

### MTS cell proliferation assay

A commercially available CellTiter 96^®^ AQueous One Solution Cell Proliferation Assay (Promega, Madison, WI, USA) 3‐(4,5‐dimethylthiazol‐2‐yl)‐5‐(3‐carboxymethoxyphenyl)‐2‐(4‐sulfophenyl)‐2H‐tetrazolium (MTS) assay was used to evaluate cell proliferation. In brief, approximately 1 × 10^4^ SK‐BR‐3 breast cancer cells were seeded in 96‐well plates and infected with Ad‐GFP or Ad‐GFP‐YB1 CTD for 48 h. Next, 20 μL of MTS reagent were added to each well, and the plate was incubated in the dark for 2 h before detection. Absorbance was measured using a Biotek Epoch Spectrophotometer (Bio Tek Instruments, Inc., Winooski, VT, USA) at 490 nm.

### Flow cytometry

The SK‐BR‐3 breast cancer cells were infected with Ad‐GFP or Ad‐GFP‐YB1 CTD vectors for 48 h for flow cytometry analysis. Then, cells were harvested by trypsinization, fixed and stained with propidium iodide (PI). Cells that demonstrated less than the diploid content of DNA were excluded from the measurement of cell percentages in each cell cycle phase. Cell cycle profile was determined with BD FACSCALIBUR flow cytometer and BD CellQuest^™^ pro software (Becton, Dickinson and Company, Franklin Lakes, NJ, USA). The experiment was independently repeated three times.

### Actin cytoskeleton staining

To analyse the actin cytoskeleton, SK‐BR‐3 breast cancer cells were infected with Ad‐GPF or Ad‐GFP‐YB1 CTD for 48 h, fixed in 4% paraformaldehyde and permeabilized with 0.1% Triton X‐100 at room temperature for 10 min. Thereafter, cells were incubated with TRITC‐phalloidin in the dark. Staining with 4′,6‐diamidino‐2‐phenylindole (DAPI) was used to visualize nuclear localization. Fluorescence microscopy was performed using the BX53 Microscope Systems (Olympus, Japan).

### Cell migration assay

Cell migration was assessed using a cell‐wounding assay. Briefly, SK‐BR‐3 cells were infected with pAd‐GPF or pAd‐GFP‐YB1 CTD vectors for 48 h and grown to 100% confluence on glass slides. Then, cells were scraped off the slides with a cell scraper and were treated with 2 μg·mL^−1^ mitomycin C (Solarbio Life Sciences, Beijing, China) and incubated in DMEM containing 10% FBS at 37 °C. The experiment was terminated 48 h later. Cells were fixed with methanol and photomicrographs of final wounds were taken for each group. Cell migration activity was expressed as the wound healing area in each field and as the percentage of control. The experiment was repeated thrice.

Next, endothelial cell transwell migration assay was performed to determine the chemotactic motility of EA.hy926 cells using Transwell^®^ Permeable Supports (Corning Incorporated, Corning, NY, USA) with 8‐μm pore size. EA.hy926 endothelial cells (1 × 10^5^ per well) were seeded in the upper chamber of a Transwell plate in DMEM with 0.5% FBS. SK‐BR‐3 cells were infected with Ad‐GFP or Ad‐GFP‐YB1 CTD vectors for 48 h, trypsinized and seeded (1 × 10^5^ cells per well) in the lower chamber in DMEM with 0.5% FBS. After 10 h, EA.hy926 cells that remained on the upper membrane surface were mechanically removed, and the number of migrated cells on the lower surface of the filter was counted under a microscope. The experiment was repeated thrice.

### Tube formation assay

To examine the effect of YB1 CTD on angiogenesis *in vitro*, tube formation assay was performed as previously described (47). Briefly, 24‐well cluster tissue culture dishes were coated with 100 μL of 10 mg·mL^−1^ chilled Matrigel (Corning, Bedford, IL, USA) and incubated for 30 min at 37 °C. EA.hy926 cells were pretreated with conditional medium from SK‐BR‐3 cells infected with Ad‐GFP or Ad‐GFP‐YB1 CTD for 24 h, and were then seeded onto solidified gels at a density of 10^5^ cells per well in 0.5 mL GFP‐CM or GFP‐YB1 CTD‐CM medium. After 8 h of incubation, total lengths of tube‐like structures in five randomly selected microscopic fields per well were determined by phase‐contrast microscopy, and quantified using image‐pro plus software 6.0 (Media Cybernetics, Inc., Rockville, MA, USA).

### Conditioned media collection

For obtaining conditioned media, SK‐BR‐3 breast cancer cells were infected with pAd‐GFP or pAd‐GFP‐YB1 CTD vectors for 24 h in complete media conditions. Thereafter, media was removed and plates were washed twice in DMEM with 0.5% FBS; and incubated for another 24 h in DMEM with 0.5% FBS. Subsequently, GFP‐overexpressing SK‐BR‐3‐conditioned media (GFP‐CM) and GFP‐YB1 CTD‐overexpressing SK‐BR‐3‐conditioned media (GFP‐YB1 CTD‐CM) were collected and stored at 4 °C until further use.

### ELISA assay

ELISA assay for quantitative estimation of VEGF in SK‐BR‐3 cell culture medium was performed using human VEGF Neobioscience ELISA Kits (Neobioscience Technology, Shenzhen, China) according to manufacturer's instructions. In brief, cell media were added into each well (100 μL per well) and incubated at 37 °C for 90 min. Then, wells were washed, and biotinylated antibody solution (100 μL per well) was added for another 60 min at 37 °C. Next, wells were washed, 100 μL of streptavidin‐HRP working solution was added into each well, and incubated in the dark at 37 °C for 30 min. Following washing, TMB (100 μL per well) was added and wells were incubated for 10 min in the dark. Finally, stop solution was added and absorbance was measured using a Biotek Epoch Spectrophotometer (Bio Tek Instruments, Inc.) at 490 nm.

### Nude mice subcutaneous injection

For *in vivo* experiments, 4–6‐week‐old female BALB/c‐nu mice were purchased from Beijing HFK Bioscience Co., Ltd (Beijing, China). All mice were bred and housed in a specific pathogen‐free environment at Hebei University Laboratory Animal Research Center, Baoding, China. All procedures performed in studies involving animals were carried out in accordance with The Code of Ethics of the World Medical Association (Declaration of Helsinki) and the ethical standards of Animal Research Ethics Committee of Hebei University. A cell suspension of 100 μL of 1 × PBS containing 5 × 10^6^ SK‐BR‐3 cells was subcutaneously injected into the right mammary fat pad of nude mice. Each experimental group consisted of six mice. Mice weight and size of the formed tumour was monitored closely; and measured every 2 days. Tumour volume was estimated according to the formula: Volume = 1/2 × *a* × *b* ^2^, where (*a*) and (*b*) represents the largest and smallest diameters of the tumour, respectively. Mice were sacrificed at day 14 after injection. Tumours were harvested, measured and photographed.

### Immunohistochemistry

Tumours from nude mice were fixed and sliced into 4‐μm‐thick sections. A manual immunohistochemical procedure was performed. In brief, after deparaffinization in xylene and rehydration in graded ethanol, endogenous peroxidase was blocked with 3% aqueous H_2_O_2_ for 25 min; and then, heat‐induced antigen retrieval was accomplished by immersing slides in 10 mm citrate buffer with pH 6 at 120 °C for 10 min. Next, slides were incubated with rabbit anti‐CD31 polyclonal antibody (Cat No. NPB1‐71663, dilution: 1 : 50, Novusbio, CO, USA) and rabbit anti‐VEGF polyclonal antibody (Cat No. 19003‐1‐AP, dilution: 1 : 50, Proteintech, Chicago, IL, USA) overnight at 4 °C. Then, slides were washed four times and processed with a ready‐to‐use HRP Anti‐Rabbit Kit (Cat No. KT1005a, Abgent Inc., San Diego, CA, USA) for 30 min at room temperature; and finally developed with 3,3′‐diaminobenzidine (DAB) as chromogen and counterstained with haematoxylin. Results were reviewed independently by two pathologists. For negative controls, slides were treated with nonimmune serum instead of the primary antibody. The expressions of CD31 and VEGF were determined using a semiquantitative visual approach. Immunostaining scoring was performed while blinded to animals' information and outcomes. Each sample was assigned to one of the following categories: 0 (0–25%), 1 (26–50%), 2 (51–75%) or 3 (76–100%). The intensity of immunostaining was determined as 0 (negative), 1 (weak), 2 (moderate) or 3 (strong). Immunostaining score formula = score of positive cell percentage × score of staining intensity.

### Statistical analysis

Data are presented as histograms of means ± SEM for three or more independent experiments. Statistical analyses were performed by spss 16.0 software using Student's *t* test or one‐way anova according to the number of groups compared. A two‐way anova and Bonferroni post‐tests were performed for the growth curve. Differences were considered significant at *P* < 0.05.

## Results

### YB1 CTD decreases SK‐BR‐3 breast cancer cell proliferation

**Figure 1:**
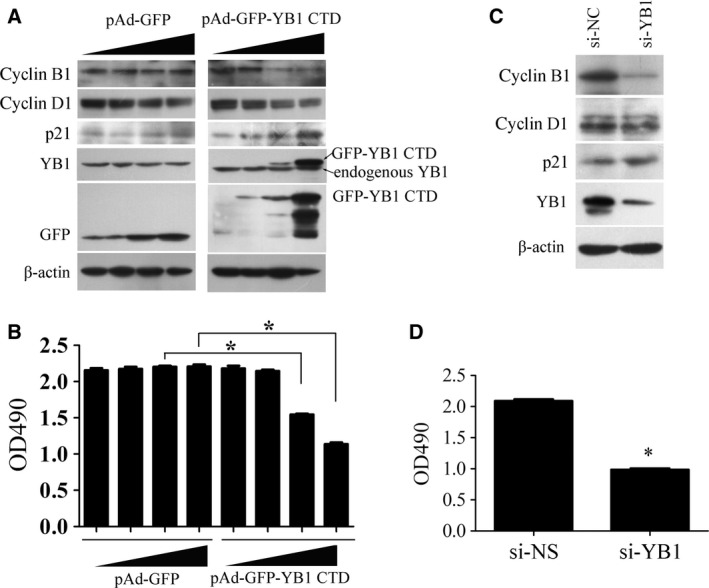
YB1 CTD decreases SK‐BR‐3 cell proliferation. (A) SK‐BR‐3 breast cancer cells were infected with different amounts of Ad‐GFP or Ad‐GFP‐YB1 CTD vectors for 48 h. Crude proteins were extracted from cells and subjected to western blotting with antibodies against cyclin B1, cyclin D1, p21, YB1 and GFP. For equal protein loading, β‐actin was used as a control. (B) SK‐BR‐3 cells were infected with different amounts of Ad‐GFP or Ad‐GFP‐YB1 CTD vectors for 48 h, cell proliferation assay was performed using the CellTiter 96^®^ Aqueous One Solution Cell Proliferation Assay Kit, and absorbance was measured at 490 nm. *P < 0.05 vs. GFP group. (C) SK‐BR‐3 cells were transfected with si‐NS or si‐YB1 for 48 h and crude proteins were extracted from the cells and subjected to western blot analysis with antibodies against cyclin B1, cyclin D1, p21 and YB1. β‐Actin was used as a control to ensure equal protein loading. (D) SK‐BR‐3 cells were transfected with si‐NS or si‐YB1 for 48 h, MTS cell proliferation assay was performed. *P < 0.05 vs. si‐NS group.

One of the aims of this study was to investigate whether YB1 CTD could regulate proliferation in breast cancer cells. For this purpose, human SK‐BR‐3 breast cancer cells were infected with different amounts of Ad‐GFP or Ad‐GFP‐YB1 CTD vectors for 48 h and western blotting and MTS cell proliferation assay were performed. As shown in Fig. 1A and B, cyclin B1 protein level decreased, p21 protein level increased, and cell proliferation activity significantly repressed in YB1 CTD‐overexpressing SK‐BR‐3 cells in a dose‐dependent manner. To further identify role of YB1 in SK‐BR‐3 breast cancer cell proliferation, endogenous YB1 was knocked down using specific siRNA targeting human YB1. Knockdown of endogenous YB1 resulted in reduced cyclin B1 protein level and decreased proliferation activity in SK‐BR‐3 breast cancer cells (Fig. 1C and D). These results indicate that overexpression of YB1 CTD repressed SK‐BR‐3 cell proliferation and proliferation‐related marker cyclin B1 expression which may due to competition for endogenous YB1.

### YB1 CTD regulates SK‐BR‐3 breast cancer cell cytoskeleton and migration

**Figure 2:**
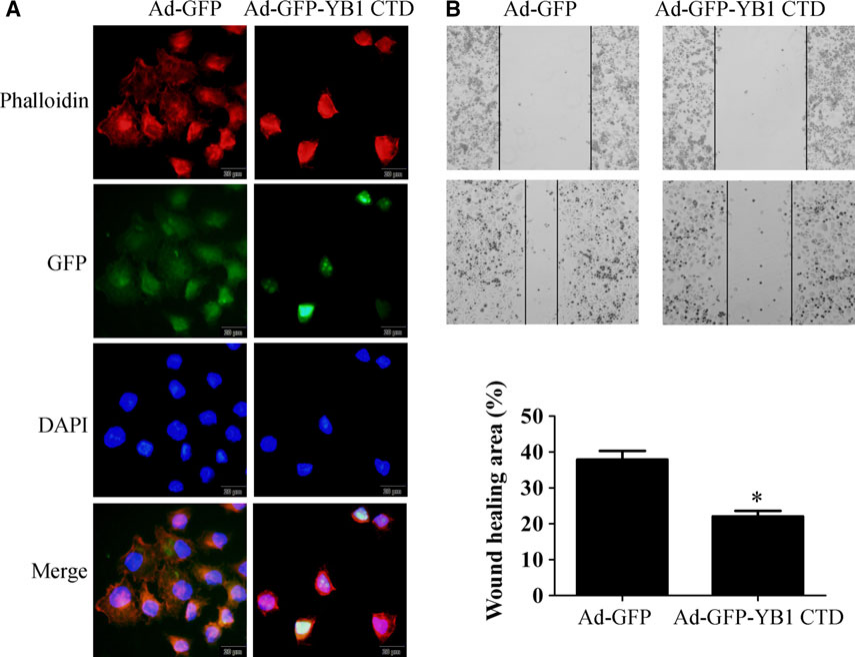
YB1 CTD regulates cell cytoskeleton and migration of SK **‐** BR‐3 cells. (A) SK‐BR‐3 breast cancer cells were infected with Ad‐GFP or Ad‐GFP‐YB1 CTD vectors for 48 h; and then, fixed and stained for F‐actin with TRITC‐phalloidin. Photographs of GFP‐tag (green), Phalloidin (red) and DAPI (purple) are presented; magnification ×400. (B) SK‐BR‐3 cells were infected with Ad‐GFP or Ad‐GFP‐YB1 CTD vectors for 48 h. Then, cells were treated with 2 μg·mL^−1^ of mitomycin C to stop proliferation and wound healing assay was performed. Migration of SK‐BR‐3 cells was photographed under a light microscope (Magnification, ×100). One representative experiment is shown (left); three independent experiments are summarized (right). *P < 0.05 vs. GFP group.

Phalloidin staining and wound healing assay were performed to evaluate the role of YB1 CTD on SK‐BR‐3 cytoskeleton and motility. SK‐BR‐3 breast cancer cells were infected with Ad‐GFP or Ad‐GFP‐YB1 CTD for 48 h. Then, cells were fixed and stained for F‐actin with TRITC‐phalloidin. As shown in Fig. 2(A), both Ad‐GFP and Ad‐GFP‐YB1 CTD‐overexpressing SK‐BR‐3 cells displayed similar actin‐rich protrusions. However, F‐actin stress fibres appeared thicker and condensed around the nucleus in Ad‐GFP‐YB1 CTD‐overexpressing SK‐BR‐3 cells, suggesting the role of YB1 CTD in actin organization. Furthermore, compared with control cells, Ad‐GFP‐YB1 CTD‐overexpressing SK‐BR‐3 cells have shown a strong reduction in microtubule extension to the cell periphery. Subsequently, wound healing assay has shown that Ad‐GFP‐YB1 CTD overexpression slightly inhibited SK‐BR‐3 cell migration ability (Fig. 2B). Collectively, these results suggest that YB1 CTD alters cytoskeleton organization and inhibits migration in SK‐BR‐3 cells.

### YB1 CTD inhibits VEGF expression and SK‐BR‐3 breast cancer cell‐induced angiogenesis *in vitro*

**Figure 3:**
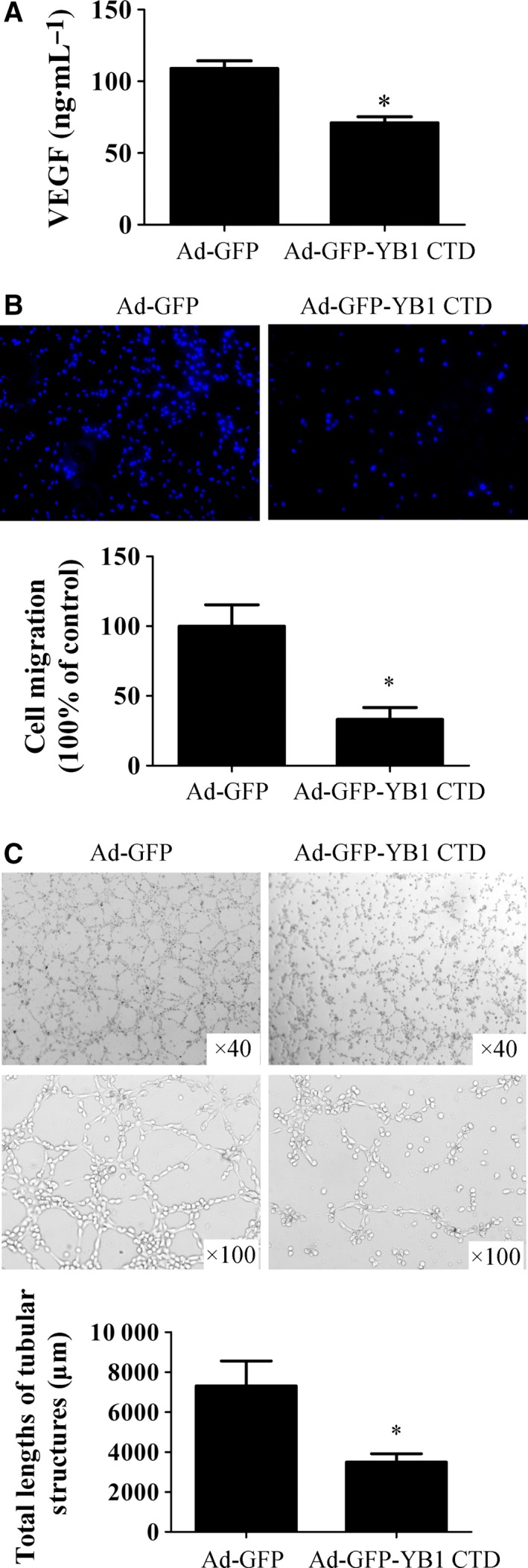
YB1 CTD inhibits VEGF expression and SK‐BR‐3 breast cancer cell‐induced angiogenesis *in vitro*. (A) SK‐BR‐3 breast cancer cells were infected with Ad‐GFP or Ad‐GFP‐YB1 CTD vectors for 24 h, media was removed, washed in DMEM with 0.5% FBS and incubated for an additional 24 h in DMEM with 0.5% FBS. GFP‐overexpressing SK‐BR‐3‐conditioned media (GFP‐CM) and GFP‐YB1 CTD‐overexpressing SK‐BR‐3‐conditioned media (GFP‐YB1 CTD‐CM) were collected, and VEGF expression in GFP‐CM and GFP‐YB1 CTD‐CM were analysed by ELISA using human VEGF ELISA kit (Neobioscience Technoloty, China). Absorbance was measured at 450 nm. *P < 0.05 vs. GFP‐CM group. (B) Transwell migration was examined using Boyden chamber assay. EA.hy926 endothelial cells (1 × 10^5^ per well) were seeded in the upper chamber of a Transwell plate in DMEM with 0.5% FBS. In the lower chamber, SK‐BR‐3 cells were infected with Ad‐GFP or Ad‐GFP‐YB1 CTD for 48 h; then, trypsinized and seeded (1 × 10^5^ cells per well) in the lower chamber in DMEM with 0.5% FBS. EA.hy926 endothelial cells that migrated through the Matrigel layer were stained and quantified after 10 h. Cell invasion was photographed under a light microscope (Magnification, ×100): one representative experiment is shown (left); three independent experiments are summarized (right). *P < 0.05 vs. GFP group. (C) EA.hy926 endothelial cells were pretreated with GFP‐CM and GFP‐YB1 CTD‐CM for 24 h. Subsequently, pretreated EA.hy926 cells (1 × 10^5^ per well) were seeded on Matrigel for 8 h. Representative photographs of tube formation are shown (upper, magnification ×40; lower, magnification ×100). Tube length was quantitated using image‐pro plus software (*n* = 5 per group). *P < 0.05 vs. GFP group.

Secreted VEGF expressions in Ad‐GFP‐YB1 CTD‐overexpressing SK‐BR‐3 cells were examined to determine the role of YB1 CTD in antiangiogenesis signalling in breast cancer cells. SK‐BR‐3 breast cancer cells were infected with Ad‐GFP or Ad‐GFP‐YB1 CTD for 48 h, and Ad‐GFP‐overexpressing SK‐BR‐3‐conditioned media (GFP‐CM) and Ad‐GFP‐YB1 CTD‐overexpressing SK‐BR‐3‐conditioned media (GFP‐YB1 CTD‐CM) were collected to detect secreted VEGF expression levels by ELISA. As shown in Fig. 3(A), Ad‐GFP‐YB1 CTD overexpression significantly decreased secreted VEGF expression levels.

Endothelial cell transwell migration assay was performed to investigate the role of YB1 CTD in SK‐BR‐3 breast cancer cell‐induced angiogenesis. For this purpose, chemotactic motility of EA.hy926 cells to Ad‐GFP or Ad‐GFP‐YB1 CTD‐overexpressing SK‐BR‐3 cells was examined. EA.hy926 endothelial cells were seeded in the upper chambers of transwell plates, while SK‐BR‐3 cells overexpressing Ad‐GFP or Ad‐GFP‐YB1 CTD were seeded in the lower chambers of transwell plates. EA.hy926 endothelial cells, which migrated through the Matrigel layer, were stained 10 h after beginning the experiment. In our study, Ad‐GFP‐YB1 CTD‐overexpressing SK‐BR‐3 cells significantly inhibited chemotatic migration of EA.hy926 cells (Fig. 3B).

Next, conditioned media GFP‐CM and GFP‐YB1 CTD‐CM were collected, and EA.hy926 endothelial cells were pretreated with GFP‐CM and GFP‐YB1 CTD‐CM for 24 h; and then, seeded on Matrigel to determine tube formation. As shown in Fig. 3(C), tube length in GFP‐YB1 CTD‐CM‐treated EA.hy926 cells were significantly shorter than GFP‐CM‐treated cells. Taken together, these results suggest that YB1 CTD inhibits SK‐BR‐3 breast cancer cell‐induced angiogenesis *in vitro*.

### YB1 CTD suppresses SK‐BR‐3 breast cancer cell‐induced tumorigenicity and angiogenesis *in vivo*

**Figure 4:**
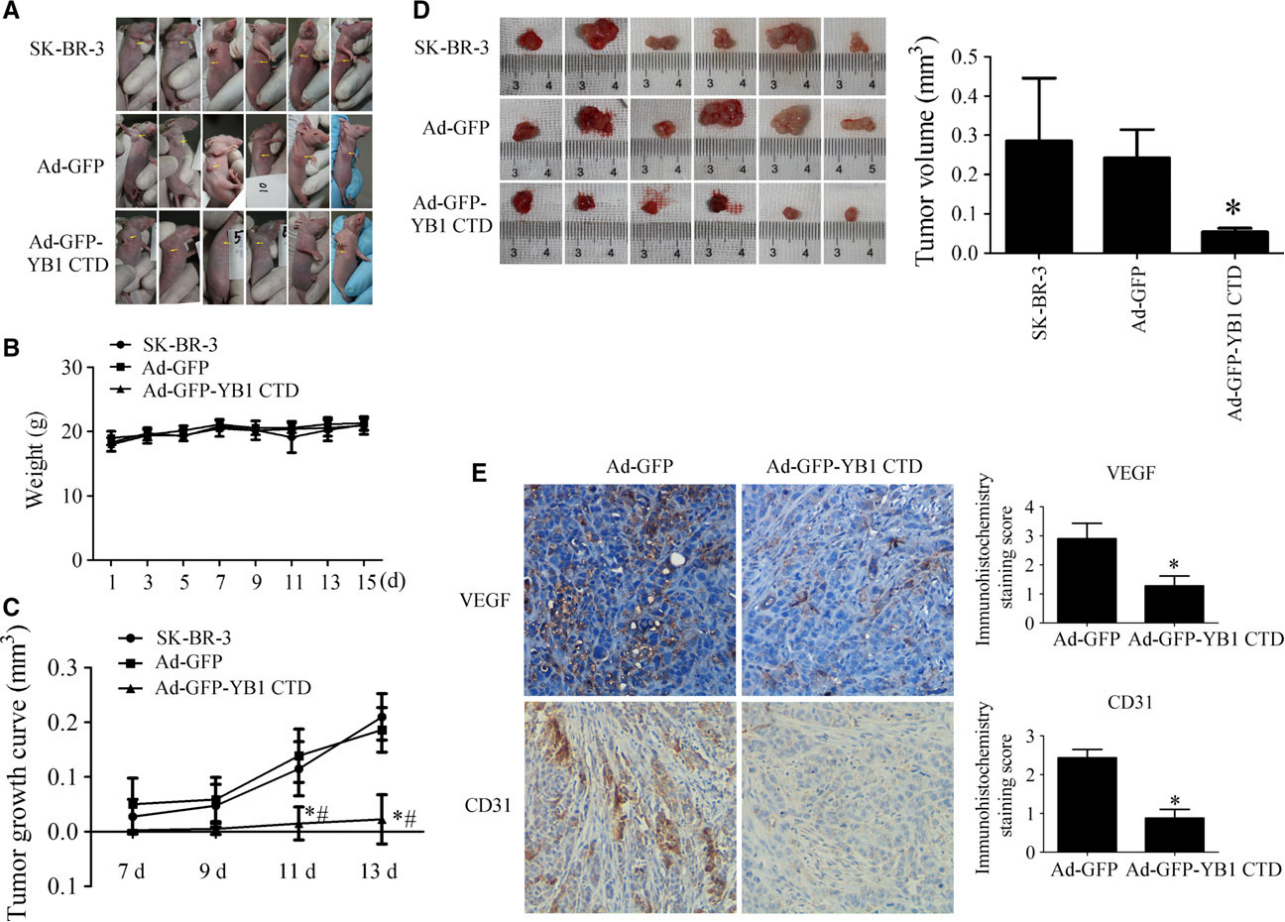
YB1 CTD suppresses SK‐BR‐3 breast cancer cell‐induced tumorigenicity and angiogenesis *in vivo*. (A) Naive SK‐BR‐3 breast cancer cells, GFP‐ or GFP‐YB1 CTD‐overexpressing SK‐BR‐3 breast cancer cells were subcutaneously injected into the right mammary fat pad of nude mice with 5 × 10^6^ cells per site. Mice were photographed 14 days after subcutaneous injection. (B) Weight of tumour‐bearing mice was monitored every 2 days after subcutaneous injection. (C) Tumour volume was monitored every 2 days after subcutaneous injection, and tumour growth curve is shown. Tumour volume was calculated by the formula: V = 1/2 × *a*(length)×*b* ^2^(width). A two‐way anova and Bonferroni post‐tests were done for the growth curve. *P < 0.05 vs. SK‐BR‐3 group. ^#^ *P* < 0.05 vs. Ad‐GFP group. (D) Tumours were harvested from mice 14 days after subcutaneous injection and photographed. Volume of tumours were measured by the formula: V = 1/2 × *a*(length)×*b* ^2^(width). *P < 0.05 vs. Naive SK‐BR‐3 group. (E) Tumours from nude mice were fixed and sliced into 4 μm thick sections. CD31 and VEGF protein levels were detected by immunohistochemical staining; magnification ×200. Left, representative photographs; right, immunohistochemical staining scores. *P < 0.05 vs. Ad‐GFP group.

SK‐BR‐3 tumour bearing nude mice model was established to further examine the antitumorigenic role of YB1 CTD. Naive Ad‐GFP‐ or Ad‐GFP‐YB1 CTD‐overexpressing SK‐BR‐3 breast cancer cells were subcutaneously injected into the right mammary fat pad of nude mice. Tumour growth was carefully monitored and size of tumour formation was measured every 2 days. Figure 4(A) shows SK‐BR‐3 breast cancer bearing mice at day 14 after injection. Weight of tumour‐bearing mice and tumour growth curve are shown in Fig. 4(B and C). General status and weight of tumour‐bearing mice have shown no significant difference among the three examined groups (Fig. 4B), while tumorigenicity and growth speed were much slower in the Ad‐GFP‐YB1 CTD group (Fig. 4C). Animals were sacrificed at day 14; and tumours were harvested, photographed and measured (Fig. 4D). Tumour volumes from naive SK‐BR‐3 and GFP groups were significantly larger than in the Ad‐GFP‐YB1 CTD group. There was no significant difference between naive SK‐BR‐3 cell and Ad‐GFP‐overexpressing SK‐BR‐3 cell groups (Fig. 4D). Taken together, these results suggest that overexpression of YB1 CTD in SK‐BR‐3 breast cancer cells attenuates SK‐BR‐3 cell ability to form tumours in nude mice.

To further identify the antiangiogenic role of YB1 CTD in SK‐BR‐3 tumour‐bearing mice, immunohistochemistry was performed to examine VEGF and CD31 expressions, as well as angiogenesis in transplanted tumour tissues. VEGF staining showed lower VEGF levels and CD31 staining showed decreased angiogenesis in Ad‐GFP‐YB1 CTD‐overexpressing SK‐BR‐3 xenograft transplants (Fig. 4E). Collectively, these findings suggest that YB1 CTD decreases VEGF levels and angiogenesis in SK‐BR‐3 tumour‐bearing mice.

## Discussion

Y‐box‐binding protein 1 is a prototypical member of the cold‐shock domain (CSD) protein family. It is a multifunctional protein with an ancient CSD domain and intrinsically complex spatial structure, which allows its interactions with transcription factors, gene promoters and mRNAs 16. These characteristics allow YB1 to participate in various cellular processes including gene transcription 14, mRNA translation 22 and DNA repair 23.

Several studies have demonstrated that YB1 expression levels are associated with aggressive tumour phenotype, poor prognosis and disease recurrence in a number of human malignancies 7, 24, 25. However, YB1 might also exhibit prodifferentiation activities depending on cellular and gene context 13. YB1 was reported to repress Chemokine ligand 5 (CCL5) transcription and promote macrophage differentiation 13. In addition, YB1 binds to Matrix metalloproteinase‐13 (MMP‐13) promoter AP‐1 site and represses MMP‐13 expression, which is essential for cancer invasion 14. In our previous study, we have demonstrated that YB1 binds to GC box in the promoter of Klf4, SM22α, p21 and cyclin D1; and promotes VSMC differentiation. Moreover, YB1‐induced differentiation and inhibited apoptosis via CTD domain in vascular smooth muscle cells 20.

In this study, we sought to reveal the role of YB1 C‐terminus in breast cancer proliferation and differentiation. Adenovirus vector harbouring YB1 CTD cDNA‐coding region, lacking the ss‐GC binding and Y‐box‐binding region, was constructed and infected into the SK‐BR‐3 breast cancer cell line. According to our results, YB1 CTD markedly decreased cell cycle‐promoting factor cyclin B1 levels and increased cyclin‐dependent kinase inhibitor p21 levels in SK‐BR‐3 breast cancer cells, which inhibited SK‐BR‐3 cell proliferation. Moreover, YB1 CTD also demonstrated the ability to disrupt cytoskeleton organization and slightly inhibits the migration of SK‐BR‐3 breast cancer cells.

Human YB1 contains three functional domain that bind to both RNA and DNA to control gene expression and regulate various cellular processes. One of the classical transcriptional control pattern of YB1 is to bind to the single‐stranded region of the promoter and either enhance or inhibit the DNA binding of other transcription factors 26. A recent study about thermodynamic characterization of YB1 and nucleic acids interactions revealed that DNA binds with the most affinity to the C‐terminal region (amino acids 130–219) of YB1 and indicated that binding of the multifunctional protein YB1 to nucleic acids regulates its biological behaviour 27. Here, we found that YB1 CTD, lacking of N‐terminal domain and CSD domain, has different functions from full‐length YB1 in SK‐BR‐3 cells, which may due to its GC‐box‐binding activity in the target gene promoter 20.

Numerous studies have shown that tumours induce sprouting angiogenesis from surrounding vasculature, which is essential for tumour growth 2, 28, 29. Our previous data have shown potential roles of YB1 CTD in proliferation and differentiation VSMCs, which are major components of vessel walls and play vital roles in vascular formation, remodelling and angiogenesis 20, 30, 31. It was also reported that YB1 represses the vascular endothelial growth factor promoter in normoxic fibroblasts 32. Another evidence revealed that YB1 can regulate tumour angiogenesis in YB1 expressing epithelial MDCK cells via release of secreted ‐modulators into the extracellular microenvironment 33. In this study, we aimed to examine the function of YB1 CTD in SK‐BR‐3 breast cancer cell‐induced angiogenesis. In order to determine the role of YB1 CTD in antiangiogenic signalling in breast cancer cells, cellular and secreted VEGF in Ad‐GFP‐YB1 CTD‐overexpressing SK‐BR‐3 cells were examined; and results demonstrated that YB1 CTD inhibited cellular and secreted VEGF expressions in SK‐BR‐3 breast cancer cells. Further experiments have shown that Ad‐GFP‐YB1 CTD overexpression in SK‐BR‐3 breast cancer cell inhibited SK‐BR‐3 cell‐induced endothelial cell transwell migration and tube formation *in vitro*. Xenograft tumour model has shown that YB1 CTD suppressed tumorigenicity *in vivo*, and decreased angiogenesis in SK‐BR‐3 tumour‐bearing mice.

In summary, in this study, we have presented the novel role of YB1 CTD in inhibiting breast cancer cell proliferation, migration and breast cancer‐induced angiogenesis *in vitro* and *in vivo*. Our results points to a new breast cancer proliferation and angiogenesis regulatory mechanism, which provides novel avenues for therapies directed against angiogenesis.

## Author contributions

J‐h.S. and B‐p.C. conceived and designed the project, J‐h.S., N‐p.C., S.W., M‐z.Z., B.W. and Y‐n.W. acquired the data, J‐h.S and N‐p.C. analysed and interpreted the data and wrote the paper.
